# CEP5 and XIP1/CEPR1 regulate lateral root initiation in Arabidopsis

**DOI:** 10.1093/jxb/erw231

**Published:** 2016-06-13

**Authors:** Ianto Roberts, Stephanie Smith, Elisabeth Stes, Bert De Rybel, An Staes, Brigitte van de Cotte, Maria Fransiska Njo, Lise Dedeyne, Hans Demol, Julien Lavenus, Dominique Audenaert, Kris Gevaert, Tom Beeckman, Ive De Smet

**Affiliations:** ^1^Department of Plant Systems Biology, VIB, B-9052 Ghent, Belgium; ^2^Department of Plant Biotechnology and Genetics, Ghent University, B-9052 Ghent, Belgium; ^3^Division of Plant and Crop Sciences, School of Biosciences, University of Nottingham, Loughborough LE12 5RD, UK; ^4^Medical Biotechnology Center, VIB, B-9000 Ghent, Belgium; ^5^Department of Biochemistry, Ghent University, B-9000 Ghent, Belgium; ^6^Centre for Plant Integrative Biology, University of Nottingham, Loughborough LE12 5RD, UK

**Keywords:** Arabidopsis, CEP5, lateral root initiation, post-translationally modified peptide, receptor kinase, XIP1.

## Abstract

Roots explore the soil for water and nutrients through the continuous production of lateral roots. Lateral roots are formed at regular distances in a steadily elongating organ, but how future sites for lateral root formation become established is not yet understood. Here, we identified C-TERMINALLY ENCODED PEPTIDE 5 (CEP5) as a novel, auxin-repressed and phloem pole-expressed signal assisting in the formation of lateral roots. In addition, based on genetic and expression data, we found evidence for the involvement of its proposed receptor, XYLEM INTERMIXED WITH PHLOEM 1 (XIP1)/CEP RECEPTOR 1 (CEPR1), during the process of lateral root initiation. In conclusion, we report here on the existence of a peptide ligand−receptor kinase interaction that impacts lateral root initiation. Our results represent an important step towards the understanding of the cellular communication implicated in the early phases of lateral root formation.

## Highlight

We identified C-TERMINALLY ENCODED PEPTIDE 5 (CEP5) as a novel, auxin-repressed and phloem pole-expressed signal assisting in the formation of lateral roots.

## Introduction

Co-ordinated positioning and development of lateral roots is central to shape root system architecture, allowing plants to adapt their below-ground organs for optimal soil exploration (De Smet, 2012; Smith and De Smet, 2012; Kong *et al.*, 2014; Tian *et al.*, 2014). Lateral root primordia are formed from approximately three pairs of xylem pole pericycle (XPP) cells arranged in neighbouring cell files that undergo asymmetric cell division and subsequently form a new organ (Dubrovsky *et al.*, 2001; Kurup *et al.*, 2005; De Smet *et al.*, 2006, 2007; Péret *et al.*, 2009; Lavenus *et al.*, 2013). In the basal meristem, close to the primary root tip and before any asymmetric cell division, a periodic transcriptional mechanism specifies pre-branch sites that are competent to form lateral roots in a regular pattern (De Smet *et al.*, 2007; Moreno-Risueno *et al.*, 2010; Van Norman *et al.*, 2013; Xuan *et al.*, 2015, 2016).

Several plant hormones have been shown to affect root architecture, among which auxin has been granted a central role (Lau *et al.*, 2008; Vanneste and Friml, 2009). In addition, a number of transcription factors and miRNAs have been shown to affect lateral root development (Satbhai *et al.*, 2015). However, several recent studies are beginning to reveal the importance of different classes of small signalling peptides during the process of lateral root development (Ohyama *et al.*, 2008; Delay *et al.*, 2013; Fernandez *et al.*, 2013, 2015; Kumpf *et al.*, 2013; Araya *et al.*, 2014; Bergonci *et al.*, 2014; Czyzewicz *et al.*, 2015). However, in Arabidopsis, very few small signalling peptides have been linked to a receptor (Murphy *et al.*, 2012; Czyzewicz *et al.*, 2013), and very few receptors involved in lateral root development have been identified (De Smet *et al.*, 2008, 2009; Kumpf *et al.*, 2013; Wierzba and Tax, 2013; Araya *et al.*, 2014; Cho *et al.*, 2014; Tabata *et al.*, 2014). Recently, the leucine-rich repeat (LRR) receptor kinases XYLEM INTERMIXED WITH PHLOEM 1 (XIP1)/C-TERMINALLY ENCODED PEPTIDE (CEP) RECEPTOR 1 (CEPR1; At5g49660) and CEPR2 (At1g72180) were proposed to act as receptors for CEP1 and other members of the CEP family (Tabata *et al.*, 2014). Both XIP1/CEPR1 and CEPR2 contain a short secretory signal peptide sequence, an N-terminal extracellular LRR receptor domain with 21 LRR repeats, a single helical transmembrane region, and a C-terminal cytoplasmic serine/threonine kinase domain. It was previously shown that a loss-of-function *xip1* mutant displays anthocyanin accumulation in the leaves, xylem-like lignification of phloem in inflorescence stems, disrupted xylem vessel formation, phloem cells sometimes located adjacent to xylem cells, and shorter inflorescence stems (Bryan *et al.*, 2012), and that the *cepr1 cepr2* double mutant displays a pleiotropic phenotype, including pale green leaves, smaller rosette leaves, shorter floral stems, anthocyanin accumulation, enhanced lateral root elongation, decreased expression of nitrate transporters, and reduced nitrate uptake activity (Tabata *et al.*, 2014). Interestingly, the *Medicago truncatula compact root architecture* (*cra2*) mutant is also affected in its root system architecture, and CRA2 was shown to be closely related to XIP1 (Huault *et al.*, 2014).

The post-translationally modified CEP family members contain an N-terminal signal peptide sequence and a C-terminal conserved CEP domain from which the mature 15 amino acid peptide is processed (Ohyama *et al.*, 2008; Delay *et al.*, 2013; Roberts *et al.*, 2013; Tabata *et al.*, 2014). Some members of the CEP family have already been shown to regulate lateral root development (Ohyama *et al.*, 2008; Delay *et al.*, 2013; Mohd-Radzman *et al.*, 2015), but in this work we functionally characterized *C-TERMINALLY ENCODED PEPTIDE5* (*CEP5*; At5g66815) in the context of lateral root initiation. Furthermore, we explored the involvement of XIP1/CEPR1 in lateral root initiation, and could show that CEP5 and XIP1 are co-expressed during early stages of lateral root initiation, and that both affect this process.

## Materials and methods

### Plant materials

The following transgenic lines and mutants were described previously: *pCEP5::NLS:GFP:GUS, CEP5* ^*OE*^ and *CEP5* ^*RNAi*^ (Roberts *et al.*, 2013), *xip1-1* and *pXIP1::GUS* (Bryan *et al.*, 2012).

### Plant growth and treatment conditions

Unless mentioned otherwise, seedlings were grown at 21 °C under continuous light (110 μE m^–2^ s^–1^ photosynthetically active radiation, supplied by cool-white fluorescent tungsten tubes, Osram) on square Petri plates (12×12cm) containing 50ml of solid half-strength Murashige and Skoog (MS) growth medium supplemented with sucrose (per litre: 2.15g of MS salts, 0.1g of *myo*-inositol, 0.5g of MES, 10g of sucrose, and 8g of plant tissue culture agar; pH adjusted to 5.7 with KOH). For peptide treatments, medium was supplemented with CEP5p^Pro^ (DFRPTTPGHSPGIGH), CEP5p^Hyp^ (DFR{HYP}TT{HYP}GHS{HYP}GIGH), or mCEP5p^Hyp^ (DFL{HYP}HT{HYP}GHV{HYP}GISH) peptide to a concentration as indicated in the text and/or figure legends. Synthetic peptides (CEP5p^Pro^, CEP5p^Hyp^, and mCEP5p^Hyp^) were obtained from GenScript (www.genscript.com/peptide-services.html?src=home), and were supplemented to growth medium with concentrations as indicated in the text and/or figure legends. For auxin treatments, medium was supplemented with indole-3-acetic acid (IAA) or 1-naphthaleneacetic acid (NAA) to a concentration as indicated in the text and/or figure legends.

### Transcriptome profiling data

The naxillin treatment transcriptome data from De Rybel *et al.* (2012) can be searched in the Lateral Root Initiation eFP Browser (bar.utoronto.ca/efp/cgi-bin/efpWeb.cgi?dataSource=Lateral_Root_Initiation) (Winter *et al.*, 2007).

### Primary and lateral root phenotyping

At the indicated time, images of plates with seedlings were taken and roots were measured using ImageJ (https://imagej.nih.gov/ij/index.html) or FIJI software (Schindelin *et al.*, 2012). For detailed staging of lateral roots, samples were cleared as described previously (Malamy and Benfey, 1997) and analysed by differential interference contrast microscopy (Olympus BX53).

### Histochemical GUS assays

For GUS (β-glucuronidase) assays, plants were put overnight in 90% acetone, then transferred to a GUS-solution {1mM X-Glc, 0.5% (v/v) dimethylformamide (DMF), 0.5% (v/v) Triton X-100, 1mM EDTA (pH 8), 0.5mM potassium ferricyanide [K_3_Fe(CN)_6_], 0.5% potassium ferrocyanide [K_4_Fe(CN)_6_], 500mM phosphate buffer (pH 7)} and incubated at 37 °C for GUS staining, and finally washed in 500mM phosphate buffer (pH 7). For microscopic analysis, samples were cleared with 90% lactic acid or as described previously (Malamy and Benfey, 1997). Samples were analysed by differential interference contrast microscopy (Olympus BX53) and stereomicroscopy (Leica MZ16). For anatomical analysis (microtome transversal sectioning) of GUS-stained roots, stained samples were processed as described previously (De Smet *et al.*, 2004).

### Real-time qRT–PCR analyses

For the analysis of *CEP5* expression, RNA was extracted by first performing an RNA extraction with TRI Reagent^®^ from Sigma-Aldrich according to the manufacturer’s protocol, followed by an extra RNA extraction procedure with the Plant RNeasy Mini kit from Qiagen according to the manufacturer’s protocol to clean up the RNA further. Next, 1 μg of total RNA was used for cDNA synthesis using the iScript cDNA synthesis kit from BIORAD according to the manufacturer’s protocol. The real-time quantitative reverse transciption–PCR (qRT–PCR) was carried out on the LightCycler 480 from Roche Applied Science with the LightCycler 480 SYBR Green I Master Mix from Roche Applied Science. The expression of *CEP5* (CCATGGACGAACCCTAAAAG and TGCCATCATCGTCTTGCTAT) was determined using at least three biological repeats and the reference genes *EEF-1α4* (CTGGAGGTTTTGAGGCTGGTAT and CCAAGGGTGAA AGCAAGAAGA) and *At2g32170* (GGACCTCTGTTGTATCA TTTTGCG and CAACCCTCTTTACATCCTCCAAAC).

### SRM analysis of the CEP5 peptide

For SRM (selected reaction monitoring) experiments, the CEP5 peptide containing an isoleucine residue with heavy, stable isotopes, NH_2_-DFRP<hydroxy>TTP<hydroxy>GHSP<hydroxy>GI(^13^C_6_^15^N)GH-COOH, was in-house synthesized by Fmoc [*N*-(9-fluorenyl)methoxycarbonyl] chemistry on a 433A peptide synthesizer (Applied Biosystems, Framingham, MA, USA). Frozen 5-day-old *35S::CEP5* seedlings were ground to a fine powder in liquid N_2_ and proteins were extracted in 50mM triethylammonium bicarbonate (TEAB) buffer containing 8M urea and the suggested amounts of protease and phosphatase inhibitors according to the manufacturer’s instructions (cOmplete protease inhibitor cocktail tablet and PhosStop phosphatase inhibitor cocktail tablet, Roche). After determining the protein concentration using the Bradford assay and diluting the protein extract twice with 50mM TEAB buffer, a total of 500 µg of protein material was filtered over a 3kDa cut-off filter (Pall Nanosep^®^ centrifugal devices, Sigma-Aldrich) to retain only peptides with masses <3kDa in the filtrate. This peptide mixture was spiked with 10 pmol of the synthetic heavy CEP5 peptide and vacuum dried. Next, the sample was re-dissolved in 2% acetonitrile (ACN) with 0.1% trifluoroacetic acid (TFA) and used for SRM analysis. SRM analysis was performed on an Ultimate 3000 RSLC nano HPLC system (Thermo Fisher Scientific, Bremen, Germany) coupled to a TSQ Vantage (Thermo Fisher Scientific). The nano-LC system was configured with a trapping column [made in-house, 100 µm internal diameter (ID)×20mm, 5 µm beads, C18 Reprosil-HD (Dr. Maisch GmbH, Ammerbuch-Entringen, Germany)] and an analytical column [made in-house, 75 µm ID×150mm, 3 µm beads, C18 Reprosil-HD (Dr. Maisch GmbH)]. The loading solvent consisted of 0.1% TFA in 2:98 ACN:H_2_O, and the nano-LC was run with 0.1% formic acid as nano-LC solvent A and 0.1% formic acid in 80:20 ACN:H_2_O as nano-LC solvent B. The needle voltage in the nano-ESI source was set at 1300V and the capillary temperature at 275 °C. A 5 µl aliquot of each sample was injected using a full loop injection. Injection was at 10 µl min^–1^ in loading solvent. After loading, the trapping column was flushed for 4min in order to pre-concentrate the components while removing buffer components, before it was put in-line with the analytical column. Compounds were eluted at 300 nl min^–1^ with an ACN gradient of 30min from 2% to 35% of nano-LC solvent B. The column was washed with 90% of nano-LC solvent B for 1min and equilibrated with nano-LC solvent A for 9.5min before analysis of the next sample. A dwell time of 120ms for each transition was applied. Seven transitions were monitored for both the heavy and the light form of the CEP5 peptide, with the doubly charged precursor as the first mass filter. Data analysis was performed through the Skyline software (MacLean *et al.*, 2010).

## Results and Discussion

### Focused transcript profiling data identifies *CEP5* as a putative regulator of lateral root development

Since the plant hormone auxin is a major regulator of primary root growth and lateral root development (Overvoorde *et al.*, 2010; Lavenus *et al.*, 2013), several transcript profiling studies based on auxin treatments have been performed in order to identify the molecular players involved (Himanen *et al.*, 2004; Vanneste *et al.*, 2005; De Smet *et al.*, 2008). However, because of the pleiotropic effects caused by exogenous auxin application, such data sets risk compromising the spatiotemporal resolution required when looking for components specific for a single developmental process. To circumvent this, we searched for putative novel early lateral root formation regulators by screening a data set obtained through a highly focused transcript profiling analysis on seedling roots treated with the synthetic molecule naxillin. Naxillin specifically induces an auxin response in the basal meristem associated with lateral root initiation through enhancing indole-3-butyric acid (IBA) to IAA conversion in the root cap (De Rybel *et al.*, 2012). Driven by the recurrent programmed cell death of the outermost lateral root cap cells, a periodic input of the converted auxin into the main root contributes to a fine-tuned mechanism that results in an evenly spaced lateral root distribution pattern (Xuan *et al.*, 2016). Importantly, through its local activity, naxillin does not display the typical pleiotropic effects of exogenous application of auxin or auxin-like molecules (De Rybel *et al.*, 2012). In order to identify novel putative early lateral root formation regulators, seedlings were grown for 72h on growth medium supplemented with the polar auxin transport inhibitor *N*-1-naphthylphthalamic acid (NPA), which prevents lateral root initiation, followed by a transfer to growth medium supplemented with naxillin to trigger the priming event synchronously in the basal meristem. In a genome-wide transcript profiling analysis, we identified *CEP5* (At5g66815) as differentially early up-regulated between non-treated and naxillin-treated seedling roots (De Rybel *et al.*, 2012) [data not shown; see Lateral Root Initiation eFP Browser (Winter *et al.*, 2007)]. The *CEP5* gene encodes a small protein of 105 amino acids and contains a conserved 15 amino acid C-terminal CEP domain that gives rise to a small signalling peptide (Ohyama *et al.*, 2008; Roberts *et al.*, 2013; Tabata *et al.*, 2014).

### *CEP5* expression is regulated by auxin

**Fig. 1.**
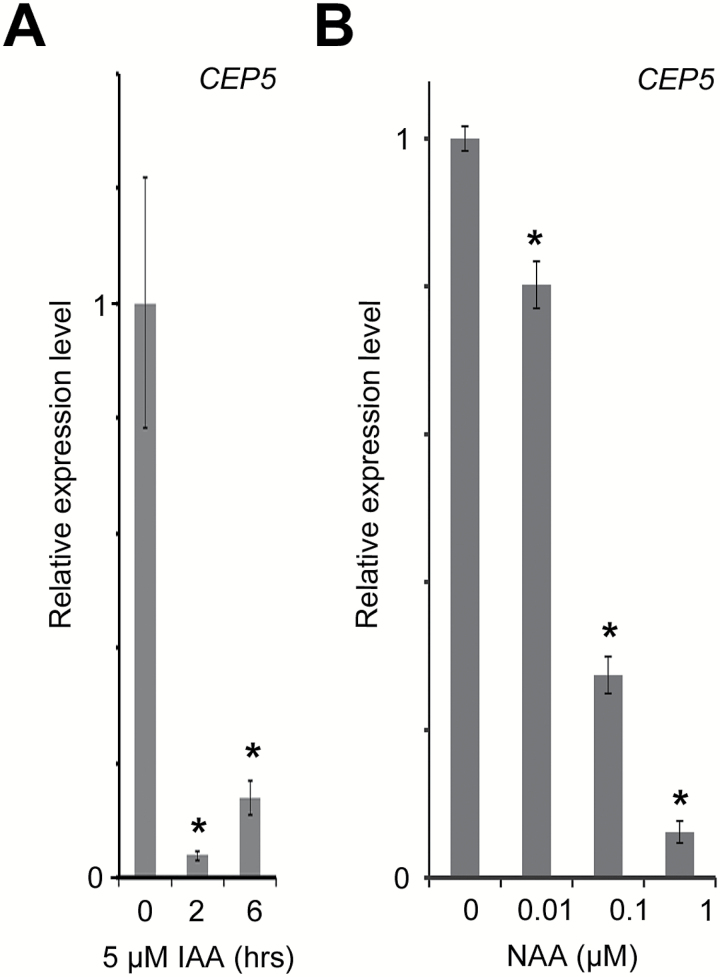
Auxin effect on *CEP5* expression. (A) *CEP5* expression in 7-day-old roots following the indicated hours of auxin (1 μM IAA) treatment in liquid medium. (B) *CEP5* expression in 5-day-old root tips of ~5mm (including the basal meristem) following 2h of auxin (NAA) treatment at the indicated concentrations. *CEP5* levels were analysed through real-time qRT–PCR. Graphs show the average ±SE of three biological replicates. *P<0.05 according to Student’s *t*-test compared with 0 μM NAA or IAA.

Since *CEP5* is transcriptionally regulated following naxillin treatment, we subsequently checked if *CEP5* expression is also auxin regulated. Treatment of wild-type roots with different concentrations of the synthetic auxin NAA or with IAA revealed that *CEP5* expression was down-regulated by auxin (Fig. 1A, B). These results suggested that *CEP5* expression is (directly or indirectly) regulated by auxin.

### *CEP5* expression is associated with early stages of lateral root development

**Fig. 2.**
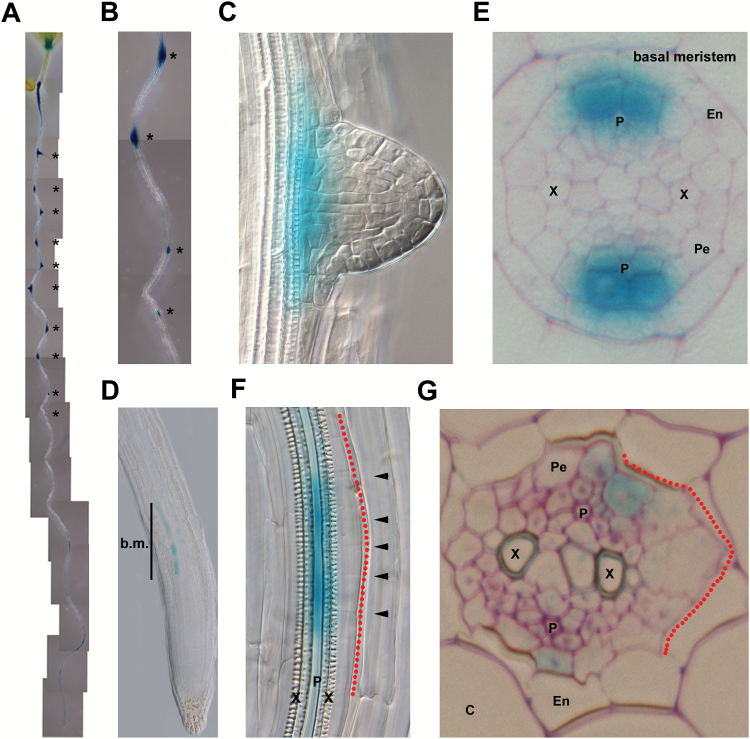
*CEP5* expression in the Arabidopsis root. Representative pictures for *CEP5* expression (monitored through *GUS* expression in a *pCEP5::NLS:GFP:GUS* transgenic line) in the root: (A) in a complete seedling (overstained for illustrative reasons), (B) in a part of the root from the seedling depicted in (A), (C) at the site of a lateral root primordium, (D) at the root apex, (E) in the basal meristem on a transverse section, (F) at a site of lateral root formation with the lateral root primordium pointing to the right (outlined with the dotted red line), and (G) on a transverse section through a lateral root primordium (outlined with the dotted red line). Seedlings are 5–6 d after germination. *, Lateral root primordium; arrowheads in (F) separate individual cells; P, phloem; X, xylem; Pe, pericycle; En, endodermis; C, cortex; b.m., basal meristem.

Based on its naxillin-regulated expression profile, CEP5 represents a candidate peptide to be involved in the early developmental steps toward lateral root development. Using a *pCEP5::NLS:GFP:GUS* reporter line (Roberts *et al.*, 2013), we observed regularly spaced patches of *CEP5* expression associated with lateral root primordia, confirming its potential involvement in this process (Fig. 2A–C). We did not detect *CEP5* expression in the primary root stem cell niche; however, *CEP5* was expressed in the basal meristem (Fig. 2D). The latter is important in the context of lateral root initiation as this region is defined as part of the oscillation zone where pre-branch sites are established by the input of auxin derived from the lateral root cap (De Smet *et al.*, 2007; Moreno-Risueno *et al.*, 2010; Xuan *et al.*, 2016). Tissue-specific analyses showed that both in the basal meristem and during early stages of lateral root development, *CEP5* was predominantly expressed in the phloem pole-associated pericycle (PPP) cells, but also—although more weakly—in the adjacent phloem (Fig. 2E–G; Supplementary Fig. S1; Supplementary Movie S1 at *JXB* online). This *CEP5* expression pattern does not overlap with the well-documented sites of high auxin response in the primary root or during lateral root initiation, which in Arabidopsis occurs in XPP cells (De Smet *et al.*, 2007). To check whether the expression pattern of *CEP5* is perturbed under conditions of altered auxin response in the XPP cells, the *pCEP5::NLS:GFP:GUS* reporter line was grown on NPA. Under these conditions, we did not observe any change in the *CEP5* expression pattern (such as radial expansion) compared with control conditions (Supplementary Fig. S1). Taken together, *CEP5* is negatively regulated by auxin and specifically expressed in the PPP cells that are closely associated with the lateral root development process, suggesting a negative correlation with auxin activity. However, what the specific cellular threshold is, is at the moment not known.

### Altering *CEP5* expression levels affects root architecture

**Fig. 3.**
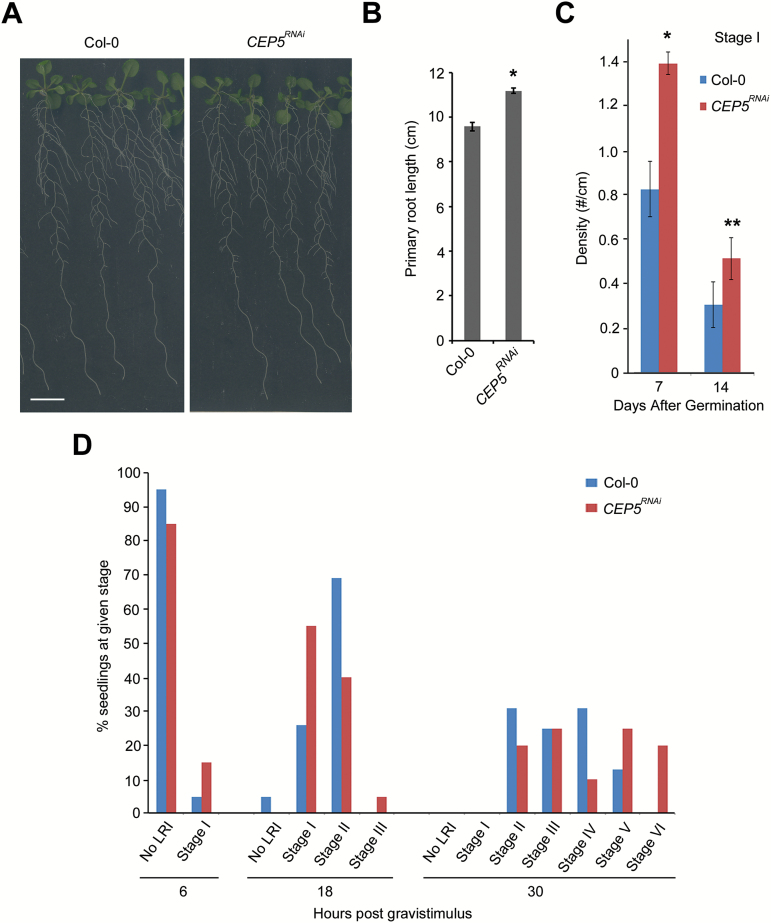
Effect of reduced *CEP5* levels on root architecture. (A) Representative picture of the *CEP5* ^*RNAi*^ line and Col-0 at 12 d after germination. (B) Quantification of the primary root length 12 d after germination (*n* ≥29). (C) Stage I lateral root primordia at the indicated seedling age (*n*=10). (D) Progression through lateral root stages at the indicated hours post-gravistimulus (*n* ≥14). Graphs in (B) and (C) show the average± SE *P<0.05 and **P<0.075 according to Student’s *t*-test compared with Col-0. Scale bar=1cm. (This figure is available in colour at *JXB* online.)

Given the spatial (appearing in common regions of the root, although not in the same cells) and temporal (being induced at the same time points) correlation of *CEP5* expression with lateral root initiation and development, we assessed if CEP5 loss of function affected this process. A *Cauliflower mosaic virus* (CaMV) *35S* promoter-driven *CEP5* RNAi knockdown line (*CEP5* ^*RNAi*^) (Roberts *et al.*, 2013) displayed a significant difference in primary root length compared with the control (Fig. 3A, B). In addition, detailed analyses of lateral root initiation in this *CEP5* ^*RNAi*^ line revealed an increased number of stage I and II lateral root primordia compared with the control (Fig. 3C; Supplementary Fig' S2). Additionally, in a root bending assay (Péret *et al.*, 2012), the *CEP5* ^*RNAi*^ line progressed faster through lateral root developmental stages than the wild type (Fig. 3D). These loss-of-function data, together with the *CEP5* expression pattern, indicate that CEP5 plays a role in early lateral root initiation events.

**Fig. 4.**
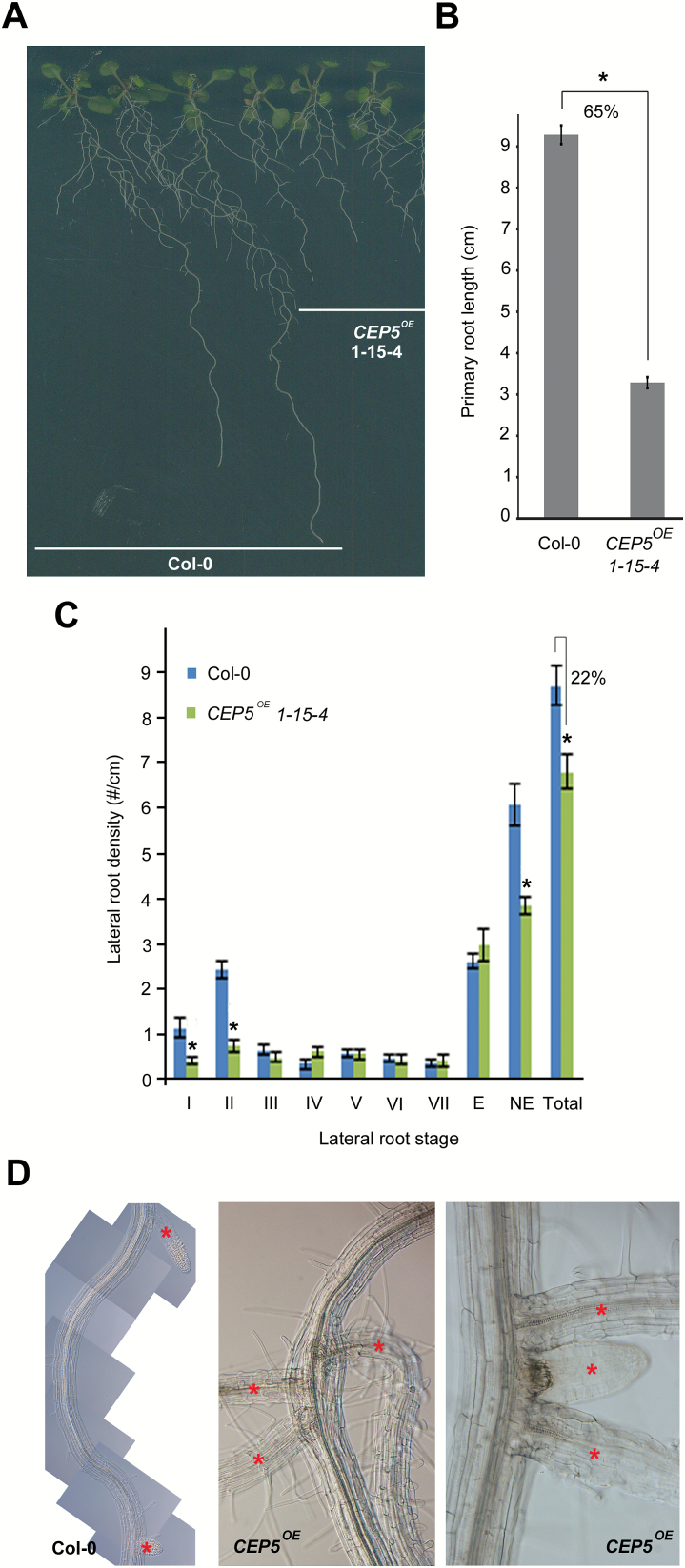
Effect of increased *CEP5* levels on primary root growth and lateral root development. (A) Representative picture of a *CEP5* ^*OE*^ line and Col-0 at 12 d after germination. (B) Quantification of primary root length at 12 d after germination. (C) Lateral root stages I–VII (according to Malamy and Benfey, 1997) in Col-0 and a *CEP5* ^*OE*^ line (*n* ≥15) at 7 d after germination. The percentage reduction in total lateral root density is indicated. E, emerged lateral roots; NE, non-emerged lateral roots; Total, sum of E and NE. (D) Regular and adjacent positioning of lateral roots in wild-type (Col-0) and *CEP5* ^*OE*^ seedlings at 14 d after germination, respectively. Asterisks in (D) indicate lateral roots. All graphs show the average ±SE of the indicated sample numbers. *P<0.05 according to Student’s *t*-test compared with Col-0. (This figure is available in colour at *JXB* online.)

Next, we analysed a line with CaMV *35S* promoter-driven constitutive overexpression of *CEP5* (*CEP5* ^*OE*^) (Roberts *et al.*, 2013), which displayed shorter primary roots (similar to other independent *CEP5* ^*OE*^ lines) as compared with the wild type (Fig. 4A, B; Supplementary Fig. S2). Furthermore, the *CEP5* ^*OE*^ line displayed a decrease in total lateral root density, with fewer non-emerged lateral roots, compared with the wild type (Fig. 4C). Detailed analyses of lateral root developmental stages showed that this was mainly due to fewer initiation events (Fig. 4C; Supplementary Fig. S3). At later stages of lateral root development, we also observed closely spaced lateral root primordia in *CEP5* ^*OE*^ lines, which we never observed as such in wild-type roots (Fig. 4D). This gain-of-function approach further suggested that CEP5 impacts root architecture, but does not exclude that this is an indirect and/or non-specific effect due to ectopic expression.

### *CEP5* gives rise to CEP5p^Hyp^

**Fig. 5.**
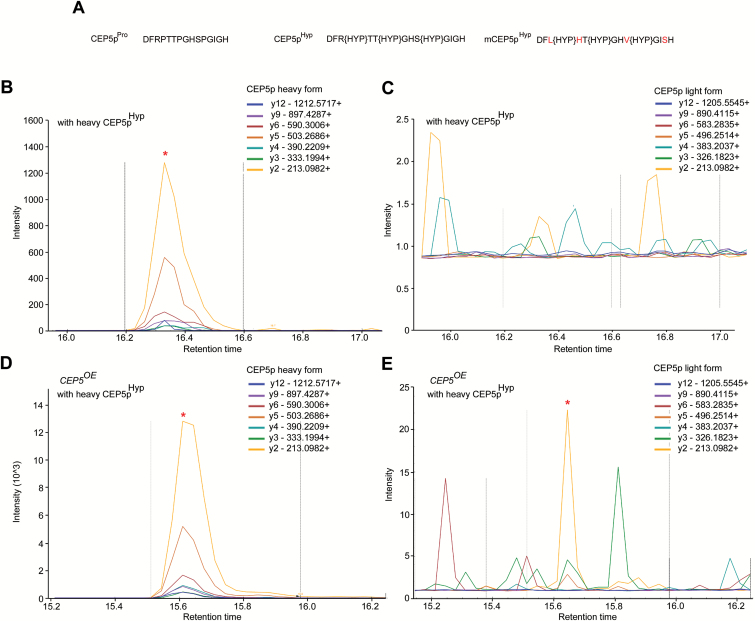
*In planta* CEP5 peptide. (A) Sequences for the synthetic variants of mature 15 amino acid CEP5: unmodified (CEP5p^Pro^), with proline hydroxylation modifications on P4, P7, and P11 (CEP5p^Hyp^), and the hydroxyprolinated mutated CEP5 sequence with four residue substitutions (R3>L, T5>H, S10>V, and G14>S; indicated in red) (mCEP5p^Hyp^). (B–E) SRM analysis of the targeted CEP5 peptide. Characteristic *y*-type of fragment ions (referred to as transitions), indicated with different colours at the top of each spectrum, were monitored. As a control, the heavy CEP5p^Hyp^ alone was analysed by SRM, and the transitions of the heavy form (B) and the light form (C) were monitored. As for the latter, no transitions could be monitored, indicating the high isotopic purity of the heavy peptide. In the *CEP5* ^*OE*^ proteome spiked with heavy CEP5p^Hyp^, both transitions for the heavy, spiked-in peptide (D) and the light, naturally occurring peptide (E) could be detected. Red asterisk, CEP5p^Hyp^.

CEP5 has a conserved C-terminal CEP domain, containing three proline residues and a predicted N-terminal signal peptide cleavage site that undergoes proteolytic processing to form a mature CEP5 peptide of 15 amino acids (CEP5p) (Roberts *et al.*, 2013; Tabata *et al.*, 2014) (Fig. 5A). However, small signalling peptides are often post-translationally modified, thereby modulating—amongst others—the ability and specificity of peptides in binding to their targets (Murphy *et al.*, 2012). In this context, it was previously shown that members of the CEP family give rise to a peptide containing hydroxyproline (Hyp) residues (Tabata *et al.*, 2014). To confirm that a 15 amino acid CEP5 peptide with three Hyp residues (CEP5p^Hyp^) (Fig. 5A) is indeed present in seedlings overexpressing *CEP5*, we performed SRM on a *CEP5* ^*OE*^ line. SRM is a mass spectrometry technique that allows detection and quantification of specific (low abundant) peptides in total protein preparations (Picotti and Aebersold, 2012). Indeed, in the *CEP5* ^*OE*^ proteome spiked with a chemically synthesized version of CEP5p^Hyp^ containing an isoleucine residue with heavy, stable isotopes, transitions for both the heavy, spiked-in CEP5p^Hyp^ and the light, naturally occurring CEP5^Hyp^ peptide could be detected (Fig. 5B–E). These results supported that a CEP5 peptide with three Hyp residues can be present *in planta*.

### Synthetic CEP5 peptide affects root architecture

**Fig. 6.**
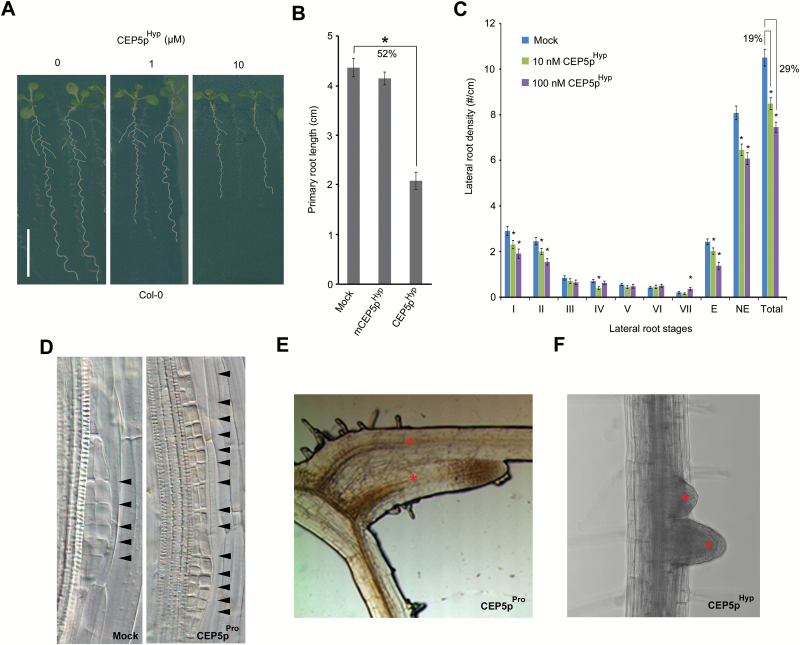
Effect of synthetic CEP5p on primary root growth and lateral root development. (A) Representative pictures of Col-0 Arabidopsis seedlings grown on the indicated CEP5p^Hyp^ concentrations for 7 d after germination. Scale bar=1cm. (B) Quantification of primary root length of Col-0 seedlings treated with 5 µM mCEP5p^Hyp^ or 5 µM CEP5p^Hyp^ compared with mock treatment at 7 d after germination (*n* ≥15 per condition). The percentage reduction in primary root length is indicated. (C) Lateral root stages I–VII (according to Malamy and Benfey, 1997) upon mock or CEP5p^Hyp^ treatment at different concentrations at 9 d after germination (data from a newly grown root part of 5-day-old seedlings transferred to CEP5p^Hyp^ for 4 d, *n* ≥32). E, emerged lateral roots; NE, non-emerged lateral roots; Total, total lateral roots. The percentage reduction in total lateral root density is indicated. (D) Pericycle cell divisions and positioning of lateral roots in mock (left) and 1 µM CEP5p^Pro^-treated Col-0 seedlings (right) (11 d after germination) (stage II primordia are shown) observed in 10 out of 149 lateral root primordia (*n*=8 seedlings), while this did not occur in the untreated wild type. (E, F) Position of lateral roots in 10 µM CEP5p^Pro^-treated seedlings 14 d after germination (E) and in 5 µM CEP5p^Hyp^-treated seedlings 12 d after germination (F). Scale bars=1cm. All graphs show the average ±SE of the indicated sample numbers. *P<0.05 according to Student’s *t*-test compared with mock. In all cases, mock refers to medium with water as used to dissolve CEP5p. Asterisk in E-F, lateral root. (This figure is available in colour at *JXB* online.)

Based on previous studies (Tabata *et al.*, 2014) and the above-described results, a synthetic CEP5p^Hyp^ peptide was generated for further analysis of CEP5 function (Fig. 5A). To assess the activity of synthesized CEP5p^Hyp^, we first analysed its effect on primary root growth, which has previously been shown to be a straightforward, although possibly non-specific, assay to test the activity of small post-translationally modified (CEP) peptides (Delay *et al.*, 2013). Indeed, seedlings grown in the presence of CEP5p^Hyp^ (also at low concentrations) displayed shorter roots compared with the mock-treated control and compared with a synthetic variant with four randomly positioned, but not very unlikely amino acid substitutions based on a BLOSUM62 substitution matrix within the 15 amino acid CEP5 peptide sequence, while retaining the Hyp residues at the same positions (mCEP5p^Hyp^) (Figs 5A, 6A, B;Supplementary Fig. S4). Next, we addressed the effect of synthetic CEP5p^Hyp^ on lateral root formation. Seedlings grown in the presence of different low concentrations of CEP5p^Hyp^ displayed a decreased total lateral root density, which is mainly due to a significant reduction in lateral root initiation events (Fig. 6C; Supplementary Fig. S3). Conversely, this did not occur in mCEP5p^Hyp^-treated seedlings (Supplementary Fig. S3). When lateral root initiation occurred, we occasionally observed regions of ectopic and/or aberrant pericycle cell divisions (observed in 10 out of 149 lateral root primordia of eight CEP5p^Pro^-treated seedlings, while this did not occur in the untreated wild type), resulting in malformed lateral root primordia or closely spaced primordia in CEP5p^Pro/Hyp^-treated seedlings, which differed from regularly spaced lateral roots in the wild type (Fig. 6D–F). Taken together, the similarities in primary and lateral root phenotypes between CEP5p treatment and *CEP5* ^*OE*^ indicate that the chemically synthesized CEP5p^Hyp^ has the same bioactivity as the overexpressed *CEP5*. These results further support a role for CEP5p^Hyp^ in lateral root initiation.

### The proposed CEP family receptor XIP1/CEPR1 regulates lateral root initiation

**Fig. 7.**
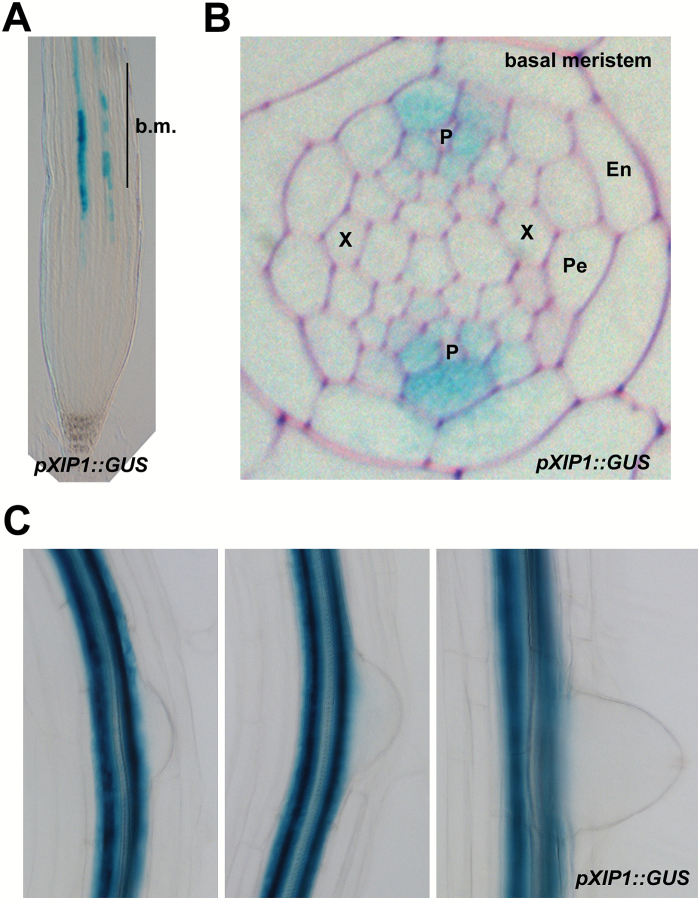
*XIP1/CEPR1* expression in the root. (A) Representative picture of *XIP1* expression in the root apex. (B) Transverse section through the basal meristem in a *pXIP1::GUS* transgenic reporter line. P, phloem; X, xylem; Pe, pericycle; En, endodermis; b.m., basal meristem. (C) Representative pictures for *XIP1* expression in different stages of lateral root development in 7-day-old seedlings. XIP1 expression was monitored through *GUS* expression in a *pXIP1::GUS* transgenic line.

**Fig. 8.**
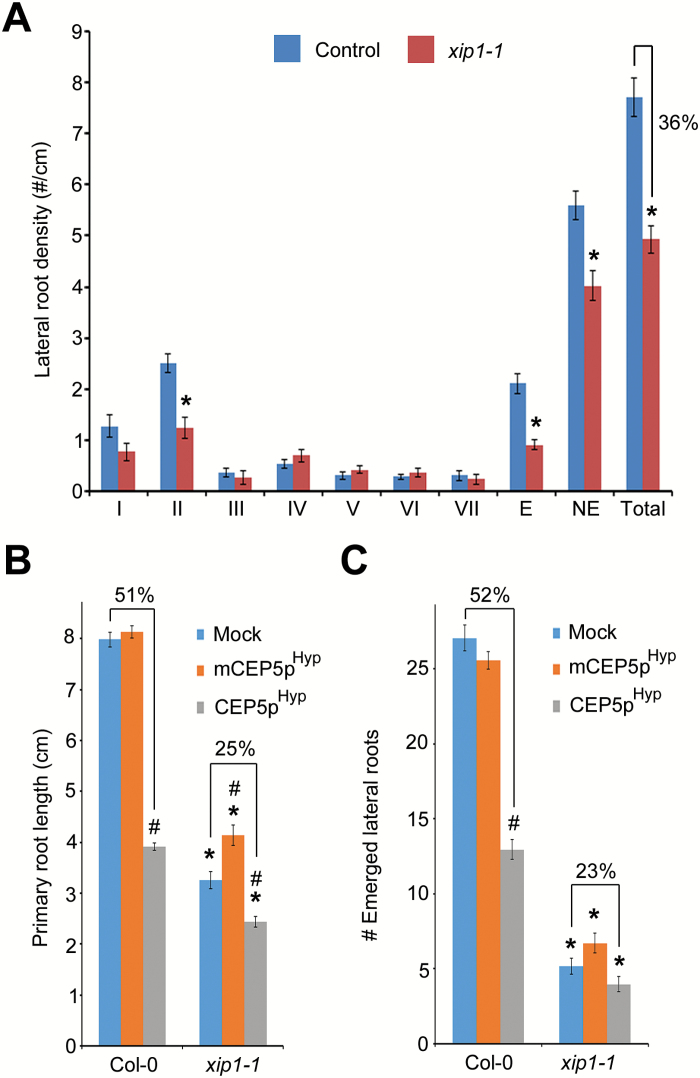
Lateral root phenotype in the *xip1-1* mutant. (A) Lateral root stages I–VII (according to Malamy and Benfey, 1997) in control and *xip1-1* at 5 d after germination (*n* ≥14). (B, C) Quantification of primary root length (B) and emerged lateral root number (C) of Col-0 and *xip1-1* seedlings treated with 1 µM CEP5p^Hyp^ or mCEP5p^Hyp^ compared with mock treatment at 10 d after germination (*n* ≥22 per condition). The percentage reduction in primary root length and lateral root number is indicated. E, emerged lateral roots; NE, non-emerged lateral roots; Total, total lateral roots. Graphs shows average ±SE. * or #, *P*<0.05 according to Student’s *t*-test compared with Col-0 or mock treatment, respectively. In all cases, mock refers to medium with water as used to dissolve CEP5p. (This figure is available in colour at *JXB* online.)

Recently XIP1/CEPR1 and CEPR2 were proposed to be the receptors for CEP peptides, including CEP5 (Tabata *et al.*, 2014). However, a role in lateral root initiation for XIP1/CEPR1 and/or CEPR2 was not yet explored. Therefore, we performed detailed analyses of a previously described *pXIP1::GUS* line (Bryan *et al.*, 2012) and we showed that *XIP1/CEPR1* is expressed in the root from the basal meristem onward (Fig. 7A), a pattern that overlaps with *CEP5* expression (Fig. 2D). Furthermore, tissue-specific analyses showed that *XIP1/CEPR1* is expressed in the phloem pole pericycle and in the adjacent phloem (Fig. 7B), confirming the overlap with *CEP5* expression (Fig. 2E), and is excluded from early stages of lateral root development (Fig. 7C), similarly to *CEP5* (Fig. 2C). This expression pattern combined with the results from Tabata *et al.* (2014) suggested that XIP1/CEPR1 could be a receptor for CEP5 in the root and therefore might take part in lateral root initiation. To explore this further, we assessed lateral root stages and density of the previously described *xip1-1* mutant (Bryan *et al.*, 2012). This revealed a reduced total lateral root density in *xip1-1* in comparison with the control, which seemed mainly due to a reduction in stage I and II lateral root primordia and—in part—to fewer emerged lateral roots (Fig. 8A; Supplementary Fig. S3), suggesting that XIP1 is a positive regulator of lateral root initiation and development.

To evaluate further an interaction between CEP5 and XIP1, we explored to what extent *xip1-1* is (in)sensitive to CEP5p^Hyp^ treatment. This revealed that, compared with the control, *xip1-1* is less or not sensitive to CEP5p^Hyp^ with respect to primary root growth (Fig. 8B) or number of emerged lateral roots, respectively (Fig. 8C). These data—together with the biochemical evidence from Tabata *et al.* (2014)—support that CEP5 and XIP1 are a peptide ligand–receptor kinase pair in the context of lateral and primary root development. However, in general, the mutant phenotypes of the genes encoding the peptide ligand and its receptor are very similar (Butenko *et al.*, 2009; Murphy *et al.*, 2012; Czyzewicz *et al.*, 2013; Kumpf *et al.*, 2013). However, in our case, the *xip1-1* root architecture phenotype is similar to that of *CEP5* ^*OE*^ or CEP5p^Hyp^-treated seedlings and opposite to that of *CEP5* ^*RNAi*^ lines (Figs 4, 6), possibly suggesting that CEP5 negatively regulates XIP1 activity (e.g. by acting as an antagonist) in the context of lateral root initiation. In this context, the fact that CEP5p^Hyp^ had no strong impact on *xip1-1* can also be interpreted as no further CEP5-mediated inhibitory effect if XIP1 is already absent (and hence fully inhibited). Alternatively, CEP5 does not exclusively act via the XIP1 receptor (or close homologues) in regulating root architecture. Furthermore, the observed lateral root phenotypes can be obtained through various mechanisms (e.g. the effect on lateral root initiation can impact development of nearby lateral root primordia), and further analyses will be required to unravel fully the developmental and biochemical mechanisms underlying CEP5 and XIP1 action.

### Conclusion

Previously, a role for CEPs in regulating aspects of root architecture, namely nitrate-dependent lateral root elongation, was proposed. Specifically, CEPs might act as root-derived ascending N-demand signals to the shoot, where their perception by CEPRs leads to the production of a putative shoot-derived descending signal that up-regulates nitrate transporter genes in the roots (Ohyama *et al.*, 2008; Delay *et al.*, 2013; Tabata *et al.*, 2014; Mohd-Radzman *et al.*, 2015). Here, we provide evidence that CEP5 may also act (probably together with XIP1/CEPR1) during lateral root initiation. Our gain-of-function and knock-down data suggest CEP5 to be part of a lateral root inhibitory mechanism. Faster lateral root development was observed in the *CEP5* ^*RNAi*^ line, while overexpression or treatment with the peptide resulted in fewer lateral root initiation events. The observed clustering of lateral roots in later developmental stages in the gain-of-function condition might be a secondary effect. Slowing down lateral root development can interfere with the timely development of auxin sources and therefore retard the draining of auxin from the main root. In turn, this might lead to higher auxin levels in the neighbourhood of existing primordia and induce ectopic and/or irregularly patterned primordia.

**Fig. 9.**
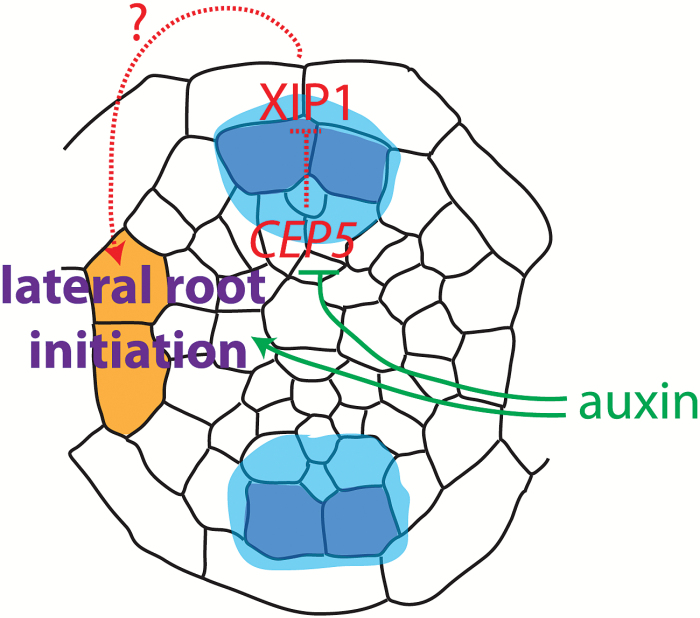
The data we have—so far—suggest that *CEP5* and *XIP1/CEPR1* are expressed in the phloem pole pericycle (PPP) cells (blue cells, with the highest expression in dark blue, and the domain with weaker, variable expression in light blue) associated with sites of lateral root formation and regulate lateral root initiation in the xylem pole pericycle (XPP) cells (orange cells) by a currently unknown mechanism (indicated by?). Overall, the CEP5 peptide appears to regulate XIP1 (activity) negatively. An auxin maximum in the XPP cells promotes lateral root initiation and possibly down-regulates *CEP5* expression in these cells. As such, the auxin minimum in the neighbouring PPP cells probably allows *CEP5* expression.

Finally, it is intriguing that a phloem-derived signal downstream of CEP5 and XIP1/CEPR1 has such an impact on lateral root initiation and development at the xylem pole (Fig. 9). So far, no mutants have been reported to show lateral root initiation at the phloem poles in Arabidopsis (and so far we have also not observed this in loss- or gain-of-function *CEP5* or *XIP1* lines) arguing for a strong and complex lateral root inhibition mechanism in this part of the root pericycle. Earlier, a cell cycle inhibitory mechanism, based on the pericycle-specific expression of *KIP-RELATED PROTEIN2* (*KRP2*), a cyclin-dependent kinase inhibitor, has been proposed as essential to allow, spatially and temporally, for lateral root initiation by repressing cell division activity in the entire pericycle except for sites of lateral root initiation (Himanen *et al.*, 2002). In the future, it will be interesting to reveal if there is any direct interaction of CEP5-dependent signalling with the control of cell cycle regulation with respect to lateral root initiation. Additionally, it will be exciting to explore alternative mechanisms on how the phloem-expressed *CEP5* affects lateral root initiation in the xylem pole pericycle cells. At the moment, however, it is not yet possible to visualize CEP5 peptide reliably *in planta* in order to evaluate possible movement to other cells and/or tissues.

## Supplementary data

Supplementary data are available at *JXB* online.

Figure S1. *CEP5* expression on a transverse section of the *pCEP5::NLS:GFP:GUS* line.

Figure S2. Analyses of *CEP5* ^*RNAi*^ and *CEP5* ^*OE*^ lines.

Figure S3. Lateral root phenotypes upon CEP5 perturbation and in *xip1-1*.

Figure S4. Bioactivity of CEP5p^Hyp^ at lower concentrations in the primary root length assay.

Movie S1. 3D reconstruction of *pCEP5::NLS:GFP:GUS* in the Arabidopsis root.

Supplementary Data
