# Supplementary material for: CEP5 and XIP1/CEPR1 regulate lateral root initiation in Arabidopsis
Source: J Exp Bot. 2016 Jun 13;67(16):4889–99. doi: 10.1093/jxb/erw231 (PMC4983111; doi:10.1093/jxb/erw231)
Supplement: Supplementary Data [file supp_67_16_4889__index.html]

CEP5 and XIP1/CEPR1 regulate lateral root initiation in Arabidopsis — CEP5 and XIP1/CEPR1 regulate lateral root initiation in Arabidopsis — Supplementary Data 

# CEP5 and XIP1/CEPR1 regulate lateral root initiation in Arabidopsis

## Supplementary Data

Data files

- supplementary\_Figures\_S1\_S4.pdf - Supplementary Data
- Supplementary\_movie\_S1.avi - Supplementary Data
