# Supplementary material for: CEP5 and XIP1/CEPR1 regulate lateral root initiation in Arabidopsis
Source: J Exp Bot. 2016 Jun 13;67(16):4889–99. doi: 10.1093/jxb/erw231 (PMC4983111; doi:10.1093/jxb/erw231)
Supplement: Supplementary Data [file supp_erw231_supplementary_Figures_S1_S4.pdf]

CEP5 and XIP1/CEPR1 regulate lateral root initiation in *Arabidopsis*

Ianto Roberts, Stephanie Smith, Elisabeth Stes, Bert De Rybel, An Staes, Brigitte van de Cotte, Maria Fransiska Njo, Lise Dedeyne, Hans Demol, Julien Lavenus, Dominique Audenaert, Kris Gevaert, Tom Beeckman, and Ive De Smet

SUPPLEMENTARY INFORMATION

Supplemental Figures

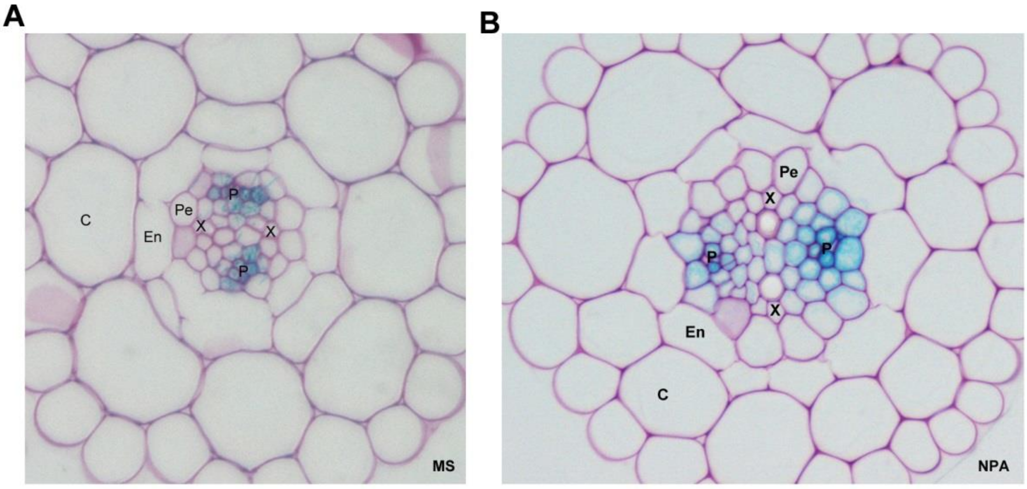

Supplementary Figure S1. ***CEP5*** **expression on transverse section.** Representative image of *CEP5* expression (monitored through *GUS* expression in a *pCEP5::NLS:GFP:GUS* transgenic line) on a transverse section through **(A)** an older part of a 5-7 day-old seedling root grown on control medium (1/2 MS) and **(B)** a 5 day-old seedling root grown on 10 µM N-1-naphthylphthalamic acid (NPA). P, phloem; X, xylem; Pe, pericycle; En, endodermis; C, cortex.

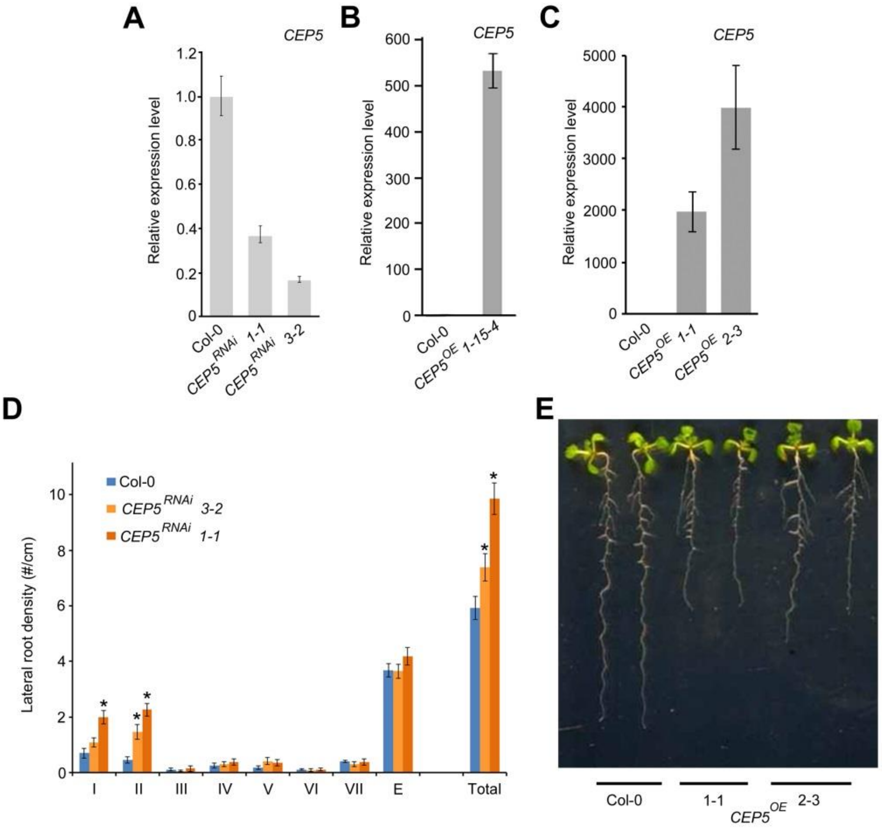

Supplementary Figure S2. Analyses of *CEP5*^{RNAi} and *CEP5*^{OE} lines. (A-C) Relative expression levels of *CEP5* in roots of independent *CEP5*^{RNAi} (A) and *CEP5*^{OE} lines (B-C) as determined by real-time qRT-PCR compared to Col-0 control roots. Graphs are average ± standard error. **(D)** Lateral root stages I to VII (according to Malamy and Benfey, 1997) in two *CEP5*^{RNAi} lines (n = 6-8) at 7 days after germination. E, emerged lateral roots; Total, sum of emerged and non-emerged. *, $p < 0.05$ according to Student's *t*-test compared to Col-0. **(E)** Representative seedlings illustrating root phenotype in independent *CEP5*^{OE} lines with relative *CEP5* expression levels shown in (C)**.**

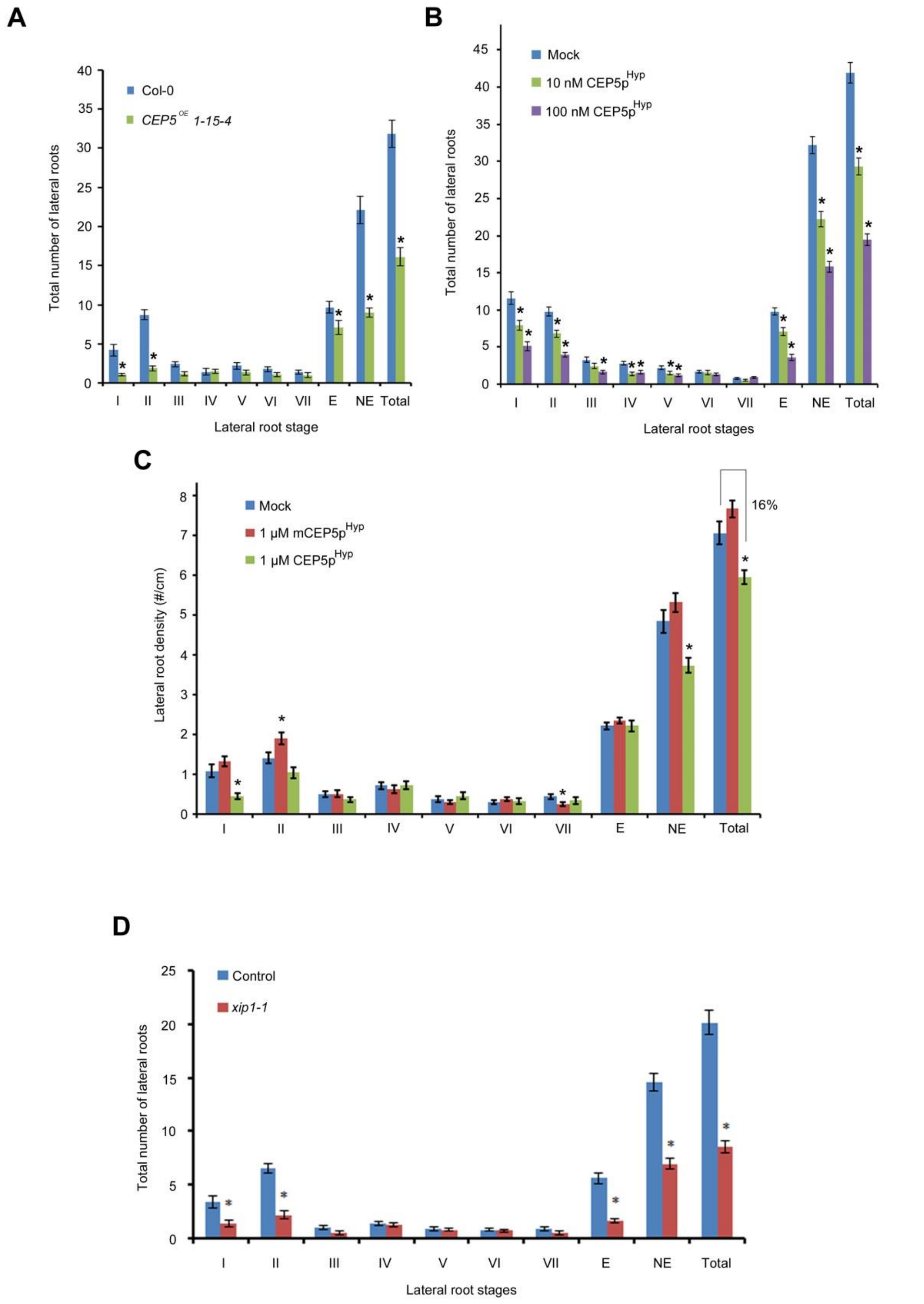

Supplementary Figure S3. Lateral root phenotypes upon CEP5 perturbation and in *xip1-1*. (A-B) Total number of lateral roots for *CEP5^OE^* (A) and CEP5p^Hyp^ treatment at indicated concentrations (B). **(C)** Lateral root density for seedlings treated with 1 μM

$CEP5p^{Hyp}$ or m $CEP5p^{Hyp}$. Lateral root stages I to VII according to (Malamy and Benfey, 1997) in *$CEP5^{OE}$* line (n ≥ 15) at 7 days after germination (A), upon mock or $CEP5p^{Hyp}$ treatment at different concentrations at 9 days after germination (data from newly grown root part of 5 day old seedlings transferred to $CEP5p^{Hyp}$ for 4 days, n ≥ 32) (B), and upon mock, $CEP5p^{Hyp}$ or $mCEP5p^{Hyp}$ treatment at 9 days after germination (data from newly grown root part of 5 day old seedlings transferred to $(m)CEP5p^{Hyp}$ for 4 days, n ≥ 23) (C). The % reduction in total lateral root density is indicated in C. In all cases, mock refers to medium with water as used to dissolve CEP5p. **(D)** Total number of lateral root stages I to VII according to (Malamy and Benfey, 1997) in Col-0 and *xip1-1* at 5 days after germination (n ≥ 14). All graphs show average ± standard error. *, $p < 0.05$ according to Student's *t*-test compared to Col-0 or mock. E, emerged lateral roots; NE, non-emerged lateral roots; Total, total lateral roots.

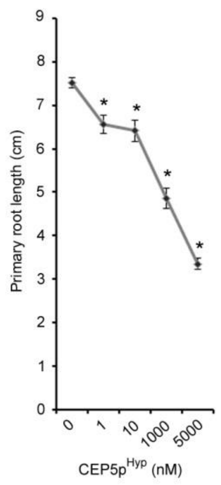

Figure S4. Bioactivity of hydroxyprolinated CEP5 (CEP5p^{Hyp}) at lower concentrations in the primary root length assay (on Col-0) at 12 days after germination (n ≥ 16 per condition). *, $p < 0.05$ according to Student's *t*-test compared to mock (medium with water as used to dissolve CEP5p).

REFERENCES

Malamy JE, Benfey PN. 1997. Organization and cell differentiation in lateral roots of Arabidopsis thaliana. *Development* **124,** 33-44.
